# *Mycobacterium tuberculosis* is the causative agent of tuberculosis in the southern ecological zones of Cameroon, as shown by genetic analysis

**DOI:** 10.1186/1471-2334-13-431

**Published:** 2013-09-13

**Authors:** Jean Paul Assam Assam, Véronique Penlap Beng, Fidelis Cho-Ngwa, Michel Toukam, Ane-Anyangwe Irene Ngoh, Mercy Kitavi, Inoster Nzuki, Juliette N Nyonka, Emilienne Tata, Jean Claude Tedom, Robert A Skilton, Roger Pelle, Vincent P K Titanji

**Affiliations:** 1 Biotechnology Unit, Faculty of Science, University of Buea, P.O. Box 63, Buea, Cameroon; 2 Laboratory for Tuberculosis Research, CANTAM TB project Biotechnology center of Nkolbisson, Faculty of Science, University of Yaoundé I, P.O. Box 337, Yaoundé, Cameroon; 3 Faculty of Medicine and Biomedical Science, University of Yaoundé I, P.O. Box 337, Yaoundé, Cameroon; 4 Biosciences eastern and central Africa -International Livestock Research Institute -Hub (BecA-ILRI Hub), P.O. Box 30709, Nairobi 00100, Kenya; 5 International Livestock Research Institute (ILRI), P.O. Box 30709, Nairobi 00100, Kenya

**Keywords:** *Mycobacterium tuberculosis* complex, MIRU-VNTR, Spoligotyping, Cameroon

## Abstract

**Background:**

Tuberculosis (TB) is a major cause of mortality and suffering worldwide, with over 95% of TB deaths occurring in low- and middle-income countries. In recent years, molecular typing methods have been widely used in epidemiological studies to aid the control of TB, but this usage has not been the case with many African countries, including Cameroon. The aims of the present investigation were to identify and evaluate the diversity of the *Mycobacterium tuberculosis* complex (MTBC) isolates circulating in two ecological zones of Cameroon, seven years after the last studies in the West Region, and after the re-organization of the National TB Control Program (NTBCP). These were expected to shed light also on the transmission of TB in the country. The study was conducted from February to July 2009. During this period, 169 patients with symptomatic disease and with sputum cultures that were positive for MTBC were randomly selected for the study from amongst 964 suspected patients in the savannah mosaic zone (West and North West regions) and the tropical rainforest zone (Central region). After culture and diagnosis, DNA was extracted from each of the MTBC isolates and transported to the BecA-ILRI Hub in Nairobi, Kenya for molecular analysis.

**Methods:**

Genetic characterization was done by mycobacterial interspersed repetitive unit–variable number tandem repeat typing (MIRU-VNTR) and Spoligotyping.

**Results:**

Molecular analysis showed that all TB cases reported in this study were caused by infections with *Mycobacterium tuberculosis (98.8%*) and *Mycobacterium africanum (M. africanum) (1.2%*) respectively. We did not detect any *M. bovis.* Comparative analyses using spoligotyping revealed that the majority of isolates belong to major clades of *M. tuberculosis*: Haarlem (7.6%), Latin American-Mediterranean (34.4%) and T clade (26.7%); the remaining isolates (31.3%) where distributed among the minor clades. The predominant group of isolates (34.4%) corresponded to spoligotype 61, previously described as the “Cameroon family. Further analysis based on MIRU-VNTR profiles had greater resolving power than spoligotyping and defined additional genotypes in the same spoligotype cluster.

**Conclusion:**

The molecular characterization of MTBC strains from humans in two ecological regions of Cameroon has shown that *M. tuberculosis sensu stricto is* the predominant agent of TB cases in the zones. Three decades ago, TB was reported to be caused by *M. africanum* in 56.0% of cases. The present findings are consistent with a major shift in the prevalence of *M. tuberculosis* in Cameroon.

## Background

Tuberculosis (TB) represents one of the most challenging threats to global human health. *M. tuberculosis* causes about 8.8 million new cases of active tuberculosis and 1.1 million deaths annually [1]. Moreover, it is estimated that over one-third of the world population has latent tuberculosis infection, which represents a huge reservoir for the disease. Exponential development of travel extends this threat worldwide [1]. Ninety-five percent of cases occur in developing countries, where the lack of proper health care systems leads to incomplete case detection and treatment. The high prevalence of HIV, which weakens the immune system, favours the spread of latent TB infection .The emergence and spread of resistance to first line TB drugs has rendered the control of the disease more difficult in sub-Saharan Africa [2].

In Cameroon, a country with 18 million inhabitants, the incidence rate of TB is estimated to have increased from 77 cases per 100,000 inhabitants in 2000 to 91 cases per 100,000 inhabitants in 2004 [3]. In 2007, about 43% of all new cases were shown to occur in HIV positive individuals. According to the National TB Control Programme (NTBCP) in 2008, 25,125 new sputum smear-positive cases were reported. Despite the implementation of the directly observed treatment short course strategy (DOTs) the incidence is still increasing [4]. One study performed 30 years ago reported that 56% of cases of TB were due to *M. africanum* strains in the West and South regions of Cameroon [5]. Another study in the West region of Cameroon showed that 42% of the *M. tuberculosis* strains collected between July 1997 and June 1998 belonged to one highly genetically-related group of strains designated the “Cameroon family” [4].

Several intervention strategies are expected to reduce the incidence of TB. In recent years, molecular typing methods have become useful tools in epidemiological studies for the control of TB, and have revealed insights into the population structure of clinical isolates in different geographical locations. Two of the most useful typing methods are spoligotyping [6] based on polymerase chain reaction (PCR) amplification of a highly polymorphic direct repeat locus in the *M. tuberculosis* genome, and mycobacterial interspersed repetitive-unit–variable-number tandem-repeat (MIRU-VNTR) [7]^,^ which uses length polymorphisms of minisatellite-like loci in the genome.

Spoligotyping targeting the direct repeat locus is a rapid, simple, and cost-effective system that allows the simultaneous detection and differentiation of *M. tuberculosis* complex (MTBC) strains and provides genotypic information [6]. It is a good indicator of strain identity and provides information about epidemiologically important clones [8]. Another advantage of spoligotyping is its ability to measure the overall diversity of *M. tuberculosis* complex strain patterns, including differences between regions and populations and the prevalence of endemic strains [9,10].

MIRU-VNTR typing is technically flexible, as sizing can be done using capillary [11,12] or gel electrophoresis [13] or non denaturing high-performance liquid chromatography [14]. It is one of the most promising PCR-based method for detecting the number of tandem repeats at a given genetic locus. Supply et al. [15] defined a set of 15 MIRU-VNTR loci for molecular epidemiological investigations and a set of 24 MIRU-VNTR loci for phylogenetic analysis of *M. tuberculosis* strains worldwide. In support of this, another study concluded that this “real-time” MIRU-VNTR genotyping approach was highly applicable for population based studies [16]. This view was reinforced by a study conducted in the Brussels region, where the authors concluded that a standardized MIRU-VNTR genotyping method could be a new reference for epidemiological and phylogenetic screening of *M. tuberculosis* strains because it is useful to confirm spoligotyping clusters or to discriminate among the isolates that they contain [17].

The aims of the present study were to identify and evaluate the diversity of the *M. tuberculosis* complex isolates using spoligotyping and MIRU-VNTR in the savannah mosaic zone (West and North West regions) and the tropical rainforest zone (Central region) in Cameroon, seven years after the last studies in the West Region and after the re-organisation of the National TB Control Programme (NTBCP). These were expected to shed light also on the transmission of TB in the country since the last studies.

## Methods

### Study population and classification of samples

The study was conducted from February to July 2009 and among 964 subjects 169 patients presented positive culture for MTBC. Seventy-one of these patients came from three centers for detection of TB (CDT) of the rainforest zone (Jamot Hospital, Mbalmayo District Hospital and Catholic Health Center of Mvolyé), 98 patients came from three CDT of the Savannah mosaic zone (Regional Hospital of Bafoussam, District Hospital of Baleng and District Hospital of Djeleng, Bamenda Regional Hospital). Sputum samples that were positive by microscopy from the Centre and West Regions were kept at +4°C and transported twice weekly to the Mycobacterium Laboratory of the Centre Pasteur du Cameroun in Yaoundé for bacteriological analysis. Samples from North-West Region were transported twice weekly to the Mycobacterium Laboratory of the University of Buea located at the Regional Hospital of Buea for bacteriological analysis.

### Ethical considerations

Ethical clearance No. 112/CNE/SE/09 was obtained from the Cameroon National Ethics Committee in Yaoundé. For inclusion in the study patients had to be aged 15 years and above, presenting with clinical symptoms of tuberculosis, and had to sign or thumbprint a written patient consent form.

### Bacteriological analysis

Each sputum sample was retested for acid-fast-bacilli (AFB) by the Ziehl Neelsen method [17], and then cultured in three Lőwenstein-Jensen (LJ) tubes, one of which was supplemented with a 0.4% solution of sodium pyruvate. The cultures were incubated at 37°C and examined weekly for growth for up to 10 weeks. Strain identification was based on the following criteria: growth rate, colony morphology, growth affinity for pyruvate, niacin production, reduction of nitrates and catalase activity.

Drug susceptibility testing was performed using the indirect proportion method on LJ medium as described by Canetti et al. [18]. The following anti-tuberculosis drugs were tested: Rifampicin (R) 40 mg/l, isoniazid (H_1_) 0.1 mg/l, Isoniazid (H_2_) 0.2 mg/l, streptomycin (S) 4 mg/l, and ethambutol (E) 2 mg/l. An isolate was considered resistant to a particular antibiotic if the number of colonies on the drug-containing medium was 1% or more of the number on the drug-free medium.

### DNA extraction

The DNA from clinical MTBC isolates was extracted using a standard protocol [19]. In brief, a minimum of four bacterial colonies was transferred to 200 μl Tris-EDTA buffer, pH 8, and heated for 15 min for 95°C. Fifty μl of lysozyme (20 mg/ml) was then added to each tube, followed by incubation overnight at 37°C. One hundred μl of SDS/proteinase K solution (containing 10 μl 20 mg/ml proteinase K and 90 μl 10% SDS), were then added and the tubes were vortexed gently and incubated for 10 min at 65°C. One hundred μl CTAB/NaCl solution (10%w/v CTAB [N-cetyl-N,N, N,-trimethylammoniumbromide] and 4.1% w/v NaCl in distilled water) was added, followed immediately by the addition of 100 μl 5 M NaCl. The tubes were then vortexed and incubated for 10 min at 65°C. Seven hundred and fifty μl of chloroform-isoamyl alcohol (24:1) was added to each tube, and the tubes were vortexed and centrifuged at 12,000 g for 5 min at room temperature. The genomic DNA present in the resulting aqueous phase was precipitated with ethanol and redissolved in 50 μl of Tris-EDTA buffer, pH 8.

### Spoligotyping

All isolates were analyzed by spoligotyping as described previously [6]. In brief, biotin-labelled PCR products from the amplification of the direct-repeat locus were hybridized against an array of 43 direct-repeat spacer oligonucleotides in a Miniblotter MN45 (Ocimum Biosolutions). The resulting hybridization signals were revealed by chemiluminescence and were visualized as profiles of discrete spots. Each spoligotype pattern was classified into a binary code. The data obtained were compared with the international SpolDB4.0 database, containing 35,925 spoligotypes from 122 countries [6].

### MIRU-VNTR genotyping

MIRU-VNTR analysis was performed with 24 published markers, and loci were PCR amplified as described previously [20], except that PCRs were done in single reactions and not multiplexed. In brief, PCR mixtures were prepared as follows: DNA (1 μl) was added to 9 μl PCR master mix containing 0.04 μl (0.4 U) of HotStarTaq DNA polymerase (Qiagen), 2 μl of Q-solution (Qiagen), 0.2 mM each of dATP, dCTP, dGTP, and dTTP (Qiagen), 1 μl of HotStarTaq 10 x PCR buffer, 1.5 to 3.0 mM MgCl_2_, 0.4 μM of unlabeled oligonucleotide and 0.04 to 0.4 μM of dye-labeled oligonucleotide (Applied Biosystems). Negative controls consisted of PCRs without template DNA. For positive controls PCRs were performed with DNA from reference strains *M. tuberculosis* H37Rv and *Mycobacterium bovis (M. bovis)* BCG P3 (Ocimum Biosolutions). The thermocycling conditions were identical for all 24 loci: 15 min at 95°C, followed by 40 cycles of 1 min at 94°C, 1 min at 59°C, and 1 min 30 s at 72°C, followed by a final step of 10 min at 72°C. PCR products were mixed with GeneScan 1200 LIZ Size Standard (Applied Biosystems) and analyzed on 3730 or 3130 Genetic Analyzers (Applied Biosystems). Sizing of the PCR fragments and assignment of the various VNTR alleles were performed using customized GeneMapper software (Applied Biosystems).

### Statistical analysis

The individual spoligotype patterns were compared with an updated in-house proprietary version of the SpolDB4 database at the Institut Pasteur de Guadeloupe, named SITVIT2 (http://www.pasteurguadeloupe.fr:8081/SITVITDemo). The allelic diversity at a given VNTR locus was calculated and generated automatically as described previously [21]. The MIRU-VNTR*plus* service (http://www.miru-vntrplus.org) was used to compare the MIRU-VNTR and spoligotyping profiles obtained in this study with reference strains for the assignment of MTBC species, lineages, and genotypes. The clustering rate was defined as described previously [21]. A strain cluster was defined as two or more patients infected by strains having identical spoligotypes and MIRU-VNTR patterns.

## Results

The study was conducted from February 21 to July 2009 of pulmonary TB that were culture positive for the MTBC were randomly selected for the study amongst 964 subjects who presented clinical symptoms of tuberculosis at the hospital study sites in the savannah mosaic zone (West and North West regions) and the tropical rainforest zone (Central region) (Table 1). The age of patients ranged from 15 to 75 years (mean age, 34 years). The female-to-male sex ratio was 1:3, with similar distribution in both ecological zones studied.

**Table 1:** Clinically suspected cases of tuberculosis for selected locations in Cameroon, and incidence of positive sputum smears

**Ecological zones**	**Regions**	**Sites**	**Number of clinically suspected cases**	**Number of positive sputum smears**
**Tropical rainforest zone**	Central	Jamot Hospital	75	39 (52%)
		District Hospital of Mbalmayo	125	27 (21.6%)
		Catholic health Centre of Mvolyé	113	09 (7.9%)
	**Subtotal**		**309**	**71 (23.9%)**
**Savannah mosaic zone**	West	District Hospital of Djeleng	130	35 (26.9%)
		Regional Hospital of Bafoussam	209	22 (10.5%)
		District Hospital of Baleng	104	22 (21.1%)
	North-West	Regional Hospital of Bamenda	220	27 (12.2%)
	**Subtotal**		**655**	**98 (14.9%)**
	**Total**		**964**	**169 (17.5%)**

### Identification and genotyping of *M. tuberculosis* strains

The spoligotyping method employed for genotyping of MTBC assigned 167 of the isolates to *M. tuberculosis* and 2 isolates to *M. africanum* out of a total of 169 cases tested.

**Figure 1:**
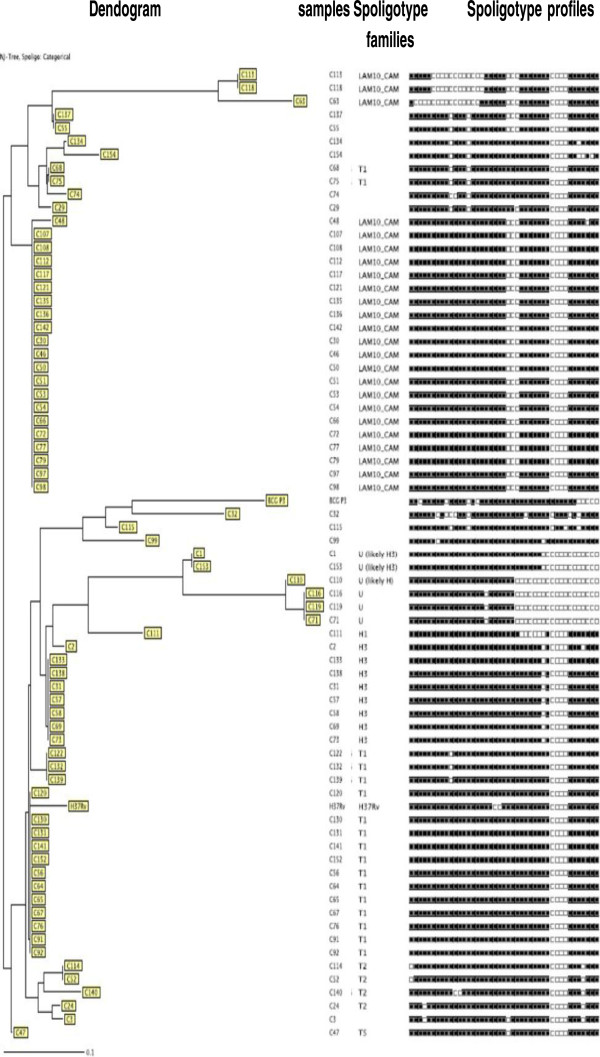
Spoligotype patterns of the 71 strains investigated in the Centre Region of Cameroon.

In the tropical rainforest zone (Centre Region), 71 isolates were subjected to spoligotyping, 17 discrete spoligotypes of *M. tuberculosis* were detected (Figure 1). A total of 59 (83%) isolates were grouped into five clusters (LAM10_CAM, Haarlem, T1, Uganda I, T2) whereas 12 (17%) presented a single spoligotype each. The 17 Spoligotypes were compared with those contained in the international spoligotyping database (SpolDB4) and it was found that 61 isolates were already described in SpolDB4 while 10 were new or unique. (C3, C29, C32, C55, C74, C99, C115, C134, C137, C154) (Table 2). The largest cluster consisted of 24 (34%) strains belonging to the Latin American-Mediterranean (LAM) family and exclusively to the one called LAM10_CAM type, 20 strains corresponding to spoligotype 61 (Cameroon family), 2 strains (ST 847), one single strain (ST 838) and another single strain (ST852). This cluster is followed by the ubiquitous T1 spoligotype for a total of 17 (23%), 12 designated in the SpolDB4 database as spoligotype 53 (Ghana strains), 3 as spoligotype 1166 and 2 as spoligotype 1475. Three other important clusters (18 strains) were found, one cluster with 8 (11%) strains belonged to the ubiquitous Haarlem family, spoligotype 50, another with 6 (8%) strains belonged to Uganda I strains with spoligotypes 450 (3 strains), 237 (2 strains) and 46 (one strain), the third cluster belonged to the ubiquitous T2 spoligotype for a total of 4 (6%) with spoligotype 848, 1056 and 853, one single strain is designated in the SpolDB4 database as spoligotype 44 (also known as the T5 family) and another as spoligotype 47 called H1 family (Table 3). The clustering rate of the spoligotyping was 0.62.

**Table 2:** Allelic diversity of the 24 MIRU-VNTR loci

	**Allelic diversity**		
**Locus**	**Centre**	**West**	**North-West**
154(MIRU 2)	0.01	0.04	0.04
424(Mtub 04)	0.24	0.41	0.22
577(ETRC)	0.57	0.59	0.28
580(MIRU 04)	0.14	0.17	0.1
802(MIRU 40)	0.74	0.71	0.69
960(MIRU 10)	0.54	0.45	0.46
1644(MIRU 16)	0.54	0.65	0.52
1955(Mtub 21)	0.59	0.37	0.39
2059 (MIRU 20)	0.62	0.56	0.66
2163b (QUB 11b)	0.74	0.73	0.63
2165(ETRA)	0.56	0.63	0.47
2347(Mtub 29)	0.47	0.51	0.56
2401(Mtub 30)	0.46	0.48	0.38
2461(ETRB)	0.71	0.71	0.51
2531(MIRU 23)	0.14	0.14	0.28
2687(MIRU 24)	0.44	0.47	0.51
2996(MIRU 26)	0.56	0.36	0.42
3007(MIRU 27)	0.56	0.57	0.65
3171(Mtub 34)	0.28	0.14	0.16
3192(MIRU 31)	0.17	0.38	0.16
3690(Mtub 39)	0.51	0.63	0.75
4052(QUB 26)	0.68	0.67	0.73
4156(QUB 4156)	0.65	0.58	0.57
4348(MIRU 39)	0.14	0.14	0.16

**Table 3:** Relative prevalence (in%) of MTBC Spoligotypes in the different regions of Cameroon

**Spoligotypes**	**Centre region**	**West region**	**North-West region**	**Average**
LAM10_CAM	33.8	30.9	37.0	33.9
Haarlem 3	11.2	4.2	3.7	6.4
T1	23.9	26.7	25.9	26.7
Uganda I	8.4	1.4	3.7	4.5
West African 1	0	1.4	0	0.4
West African 3	0	0	3.7	1.2
Orphan	14.1	22.5	14.8	17.1
T2	5.6	4.2	3.7	4.5
LAM1	0	2.8	0	0.93
T5	1.4	2.8	0	1.4
TUR	0	0	3.7	1.2
Haarlem 1	1.4	5.6	3.7	3.5

**Figure 2:**
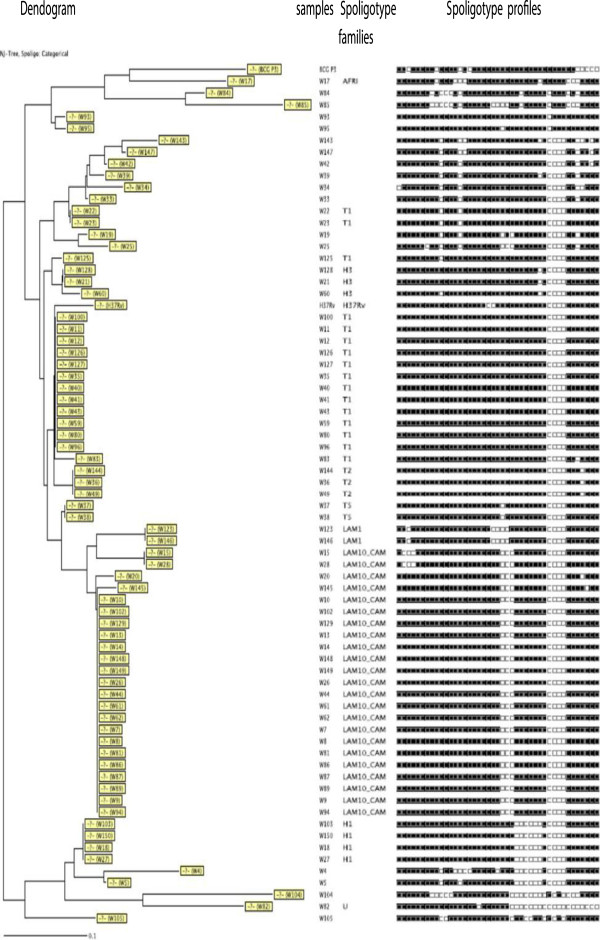
Spoligotype patterns of the 71 strains investigated in the West Region of Cameroon.

**Figure 3:**
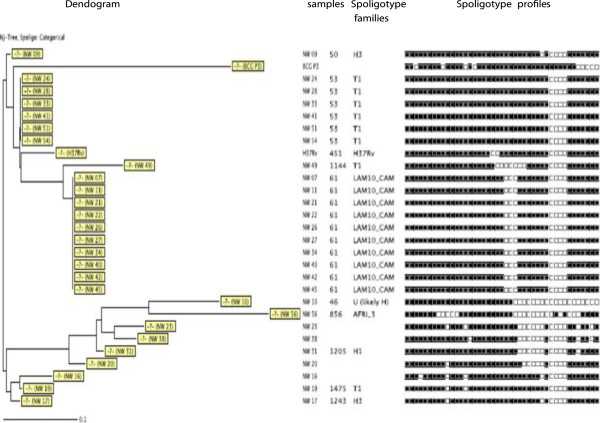
Spoligotype patterns of the 27 strains investigated in the North-West Region of Cameroon.

In the savannah mosaic zone (West region and North-West region), spoligotyping showed that 25 patterns were detected among the 98 isolates. A total of 73 (74.4%) isolates were grouped into 7 clusters (LAM10_CAM, Haarlem3, T1, T2, T5, LAM1, Haarlem1), whereas 25 (25.6%) presented a single spoligotype. The 98 isolates were compared with those contained in the international spoligotyping database (SpolDB4). A total of 78 isolates were already described in SpolDB4 while 20 were new and unique (W4, W5, W19, W25, W33, W34, W39, W42, W84, W85, W93, W95, W104, W105 W143, W147, NW 23, NW 38, NW 20, NW 16) (Table 3) and (Figures 2 and 3). Table 3 summarizes the distribution of the spoligotypes identified in the present investigation. It can be seen that the largest cluster of 33 (33.6%) belonged to the Latin American-Mediterranean (LAM) family and exclusively to the type called LAM10_CAM. Other prominent strains included the Cameroon family (spoligotype ST 61), the Ghanian and Harlem spoligotypes. One single strain is designated in the SpolDB4 database as spoligotype 450 called Uganda I family and another as spoligotype 332 called West African 1 family (Table 3). The clustering rate of the spoligotyping was 0.52.

MIRU-VNTR analysis was performed in both ecological zones on all the 169 *M. tuberculosis* isolates by using 24 published markers [13], which included 12 MIRU, 3 ETR, 7 QUB and 2 VNTR loci. The 169 isolates were all distributed into unique patterns (100%). The clustering rate of the MIRU-VNTR was 0.014 for the tropical rainforest zone and 0 for the savannah mosaic zone. The allelic diversity (*h*) differed for the individual loci, ranging from 0.01 to 0.75 for all zones investigated. The MIRU40, QUB 26 and QUB11b loci showed the highest discriminatory power (*h* = *0.74*), ETRB, QUB26, QUB4156 also showed the high allelic diversity (*h = 0.6 - 0.68*), and five other loci (MIRU 2, MIRU4, MIRU 23, MIRU31, and MIRU39, Mtub 34) showed low discriminating power (*h* < 0.2). The other supplemental locus had a relatively high allelic diversity (*0.2 < h <0.6*) (Table 2).

**Figure 4:**
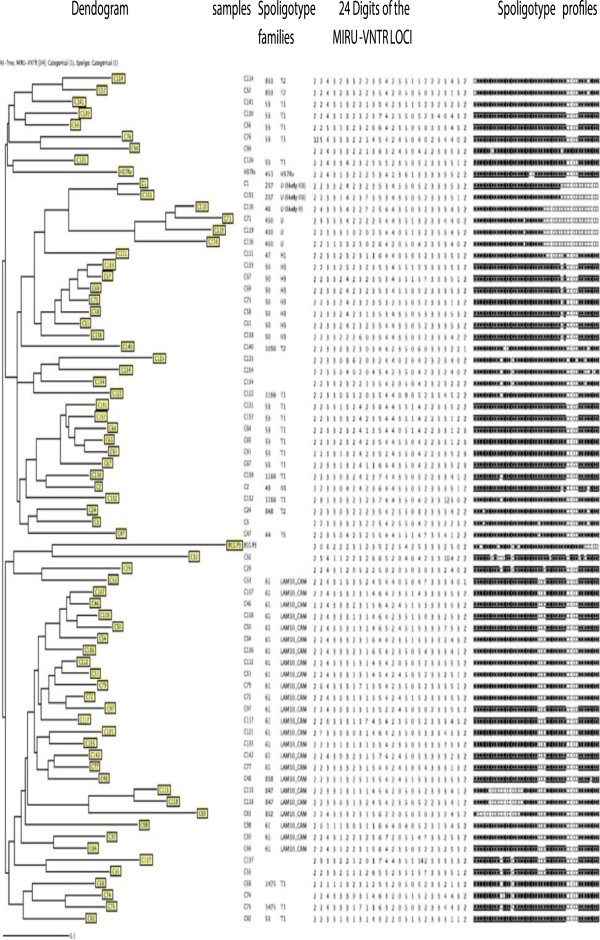
Spoligotype and 24 loci MIRU-VNTR typing patterns of the 71 strains investigated in the Centre Region of Cameroon.

**Figure 5:**
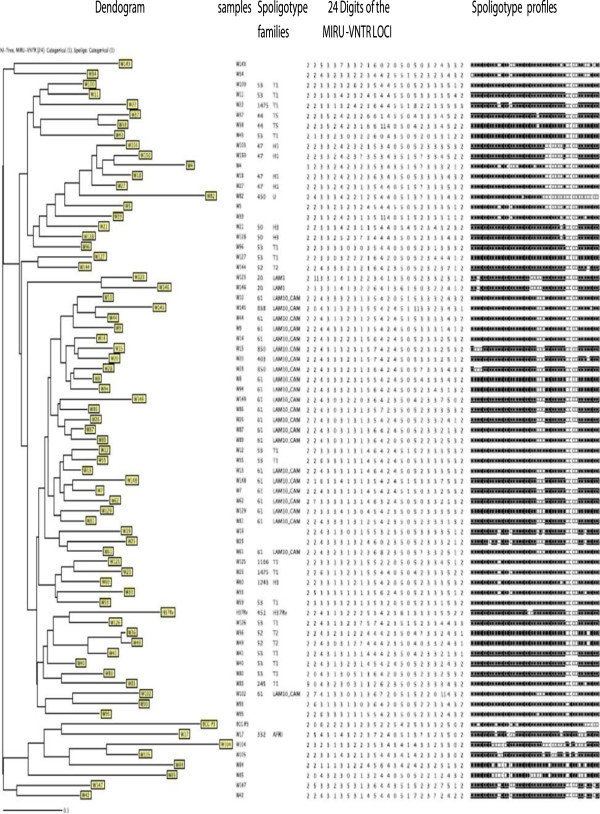
Spoligotype and 24 loci MIRU-VNTR typing patterns of the 71 strains investigated in the West Region of Cameroon.

**Figure 6:**
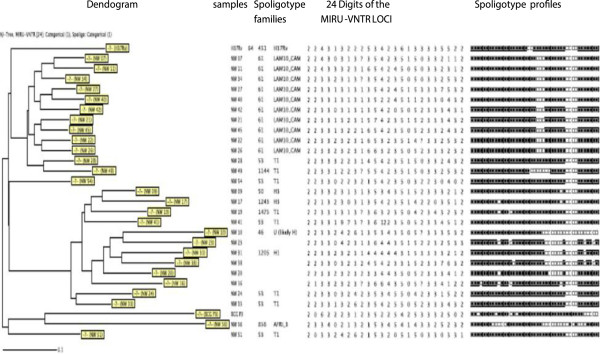
Spoligotype and 24 loci MIRU-VNTR typing patterns of the 27 strains investigated in the North-West Region of Cameroon.

The combination of the spoligotyping and MIRU-VNTR data revealed a further resolution of some of the clustered isolates identified by spoligotyping alone. (Figures 4, 5 and 6).

As earlier reported, both resistance to single first line drugs and multidrug resistance phenotypes were detected in all the ecological zones studied [22], the resistance profile compared with types of spoligotypes in the tropical rainforest zone revealed a resistance of 21% to INH. 15.7% to RIF and 15.7% to SM for LAM10_CAM (ST 61). For the same spoligotype we obtained an MDR rate of 15.7% and 5.26% for monoresistances. For the Ghanaian family, 16.6% were MDR and 8.3% were resistant to at least one drug. In the Savannah mosaic zone for the LAM10_CAM, 8.3% were resistant to INH and 4.1% to SM. For the Harlem3, 12.5% were resistant to RIF, 50% were resistant to RIF for the Uganda family, 20% of the T1 family was resistant to SM, 50% of the T2 family was resistant to both INH and RIF, only one new spoligotype presents resistant to INH. When these prevalence rates of the spoligotypes were compared with the phenotype result no significant correlations were observed. Also A comparative analysis of the 2 ecological zones did not show any correlations between the ecological zone and the distribution of spoligotypes (Table 4).

**Table 4:** Comparative analysis of the 2 ecological zones

**Distribution of the sample collected**		**Tropical rain forest zone**	**Savannah mosaic zone**	**P-value**
		**(Central region)**	**(West and North West regions)**	
	Number of clinically suspected cases	309	655	/
	Number of positive sputum smears	71	98	0.0022
**Relative prevalence of MTBC Spoligotypes**	LAM10_CAM	24	33	0.98
	Haarlem 3	8	5	0.137
	T1	17	23	0.94
	Uganda I	6	4	0.324
	Orphan	10	20	0.282

## Discussion

The main aims of the present investigation were to determine the etiologic agent of tuberculosis and its genetic diversity in Cameroon, 7 years after it was first identified and during which a national anti-tuberculosis programme had been instituted. In this study we show that *M. tuberculosis* sensu stricto is the main cause of tuberculosis among patients in two ecological zones covering three administrative regions of Cameroon.

Of the samples tested Spoligotyping and MIRU-VNTR identified 167 strains (98.8%) of *M. tuberculosis* and two strains (01.2%) of *M. africanum*. In the 1970s, most reported cases of TB in Cameroon were caused by *M. africanum*[5]. Our results indicate a shift in type of infectious agent *M. tuberculosis* versus *M. africanum* in Cameroon. A similar study conducted by Niobe-Eyangoh et al. [4] suggested that the decreasing trend observed cannot be attributed to identification bias, but probably reflects a genuine regression of *M. africanum* (from 56 to 9%) as the etiologic agent of TB in Cameroon during the last 3 decades. Studies by Simonet et al. [23] and Ledru et al. [24] also showed a decrease of *M. africanum* responsible for human tuberculosis cases in Burkina Faso. However, the factors that contribute to the reduction of this species have not been unraveled. Conceivably, the implementation of the Direct Observed Treatment strategy (DOTs) in Cameroon and our use of more sensitive and specific genotyping methods could account at least in part for the differences observed.

The molecular investigation of the Cameroonian strains by spoligotyping did not show the specific signature of *M. bovis* despite the high prevalence of bovine TB among cattle [25]. This result is in agreement with published findings in Cameroon and Burkina-Faso showing the absence of, or an extremely low prevalence of, bovine TB among humans [4,24,26]. The low rate of human pulmonary TB caused by *M. bovis* in our study could be explained by different factors: (i) a high number of the *M. bovis* infections are responsible for extrapulmonary TB cases and in our study the TB cases were essentially pulmonary cases or (ii) pulmonary TB due to *M. bovis* may be more frequent in rural areas [26]. However, the majority of the patients in our study were from urban areas (Yaounde, Mbalmayo, Bafoussam, and Bamenda).

The comparison of spoligotyping from the 2 ecological zones investigated with the International Spoligotyping database showed that the three major clusters belong to major clades of *M. tuberculosis* (LAM10_CAM: 34%; and T1: 27%; Haarlem: 7%), the remaining isolates (32%) where distributed among the minor clades. This study demonstrated that LAM10_CAM is a dominant strain just as was the case in Tanzania [27]. However, in Harare and Zimbabwe [27], LAM11 was predominant indicating that the prevalence rates of various LAM sub-families might vary among different region of Africa, and that TB and that TB epidemics might be diverse and localized. The success of the LAM family in particular in this community is intriguing and needs to be followed up in larger population-based studies.

Although spoligotyping suggested some clustering of isolates (Table 3) further analysis using the MIRU-VNTR resolved the apparent clusters into distinct genotypes. This is not surprising considering the high discriminating power of the latter method. It is therefore recommended, that wherever possible, both methods should be employed to give a clearer picture of the genetic diversity. Although a previous study in Yaoundé [28] had suggested a preponderance of drug resistant phenotypes among the LAM10_ CAM family, we found no such correlations in the present study.

The clustering rate obtained from the two methods used was very different: 0.52 to 0.62 for the spoligotying and 0 for the MIRU-VNTR. This discrepancy is probably attributable to the small size of the convenience sample employed. A population based study with a larger randomly selected sample is indicated to determine the status of TB transmission in the studied area.

## Conclusions

Our investigation of genetic polymorphism of *M. tuberculosis* complex strains from humans in two ecological regions of Cameroon has shown *M. tuberculosissensu stricto* to be the predominant agent of TB cases, with the preponderance of the LAM10 family genotype. Further studies to monitor the evolution of the dominant genotypes in the country and region are indicated as essential to the ongoing anti-TB campaign.

## Competing interests

The authors declare that they have no competing interests.

## Authors’ contributions

JPAA carried out many of the experiments as a PhD student, participated in field work and drafted the manuscript; VBP participated in field work and in the conception, design, supervision of the experiments, analysis of data and revision of the manuscript; FCN participated in field and laboratory work and in the conception, design, supervision of the experiments, analysis of data as well as in the drafting and revision of the manuscript; MT participated in field work and in the supervision of the experiments and revision of the manuscript; RAS and RP participated and supervised molecular analysis at the BecA-ILRI Hub, Nairobi, Kenya, analysis of data and revision of the manuscript; AAIN participated in the field work and in the revision of the manuscript; MK and IN assisted JPAA at ILRI, Nairobi- Kenya; JJ assisted JPAA in the field and on the laboratory bench; ET assisted JPAA in the field and on the laboratory bench; JCT assisted JPAA in the field and on the laboratory bench; VPKT was the overall supervisor and chief designer of the project. All authors read and approved the final manuscript before submission.

## Pre-publication history

The pre-publication history for this paper can be accessed here:

http://www.biomedcentral.com/1471-2334/13/431/prepub
